# Flower-like superstructures of AIE-active tetraphenylethylene through solvophobic controlled self-assembly

**DOI:** 10.1038/srep42898

**Published:** 2017-02-23

**Authors:** Mina Salimimarand, Duong Duc La, Mohammad Al Kobaisi, Sheshanath V. Bhosale

**Affiliations:** 1 School of Science, RMIT University, GPO Box 2476, Melbourne, Vic. 3001, Australia

## Abstract

The development of well-organized structures with high luminescent properties in the solid and aggregated states is of both scientific and technological interest due to their applications in nanotechnology. In this paper we described the synthesis of amphiphilic and dumbbell shaped AIE-active tetraphenylethylene (TPE) derivatives and studied their self-assembly with solvophobic control. Interestingly, both TPE derivatives form a 3D flower-shape supramolecular structure from THF/water solutions at varying water fractions. SEM microscopy was used to visualise step-wise growth of flower-shape assembly. TPE derivatives also show good mechanochromic properties which can be observed in the process of grinding, fuming and heating. These TPE derivative self-assemblies are formed due to two main important properties: (i) the TPE-core along with alkyl chains, optimizing the dispersive interactions within a construct, and (ii) amide-linkage through molecular recognition. We believe such arrangements prevent crystallization and favour the directional growth of flower-shape nanostructures in a 3D fashion.

Supramolecular self-assemblies of small aromatic organic π-conjugated molecules have attracted enormous attention of researchers due to their applicability in optoelectronics, chemosensors, nanotechnology, biotechnology and biomedicines^1^,^2^,^3^,^4^,^5^,^6^. Where non-covalent interactions (hydrogen-bonding, π-π interaction, van der Waals forces and electrostatic attraction) play important roles in the creation of controlled 1, 2 and 3 dimensional superstructures, such as nanowires, nanotubes, nanoribbons, nanobelts, nanosheets, spheres, capsules, and nano/micro-flowers from small aromatic π-conjugated functional molecules^7^,^8^,^9^,^10^,^11^,^12^.

The massive morphological changes of supramolecular assemblies have been widely investigated, however, flower-like assemblies of small organic molecules are rare. During the past decade, flower-shaped inorganic^13^,^14^,^15^,^16^,^17^/organic^18^,^19^,^20^,^21^,^22^,^23^,^24^ assemblies gained the attention of researchers due to their potential applications in the various fields such as catalysis, superhydrophobics, explosives detection, magnetic materials, biomedical and optoelectronic materials. In this regards, Nakanishi and co-workers reported the formation of organic flower-shaped morphology *via* self-organization of functionalised C_60_ derivative^25^. Furthermore, few examples for the construction of organic flower-like nanostructures from diphenylalanine dipeptide and DNA also reported^13^,^14^,^15^,^16^,^17^,^18^,^19^,^20^,^21^,^22^,^23^,^24^. Very recently, we reported flower-like structures assemblies of naphthalene diimide^7^ appended phosphonic acid with melamine from aqueous medium through strong hydrogen-bonding and electrostatic interaction^26^.

However, these assemblies suffer from drawbacks where aggregation-caused quenching (ACQ) was observed^27^, which is encountered in most conventional fluorophores used to form these assemblies. ACQ occurs due to the formation of excimers through π-π stacking of fluorescent molecules providing non-radiative relaxation pathways of the excited states. Secondly, the organic flower-shaped morphology field is in its infancy and has not been explored with respect to AIE-active small organic molecules.

To further explore this idea, tetraphenylethylene (TPE) was used due to its aggregation induced emission (AIE) properties^28^, contrary to the ACQ-effect^27^ of most commonly used small molecules in flower assembly. It is a non-planar, propeller shaped molecule, which is easily prepared with excellent yield. The AIE-effect mechanism is explained by the restriction of the intramolecular rotations (RIR) which restrict the non-radiative relaxation of the molecules *via* mechanical and thermal pathways, hence making the electronic emissive relaxation the preferred, highly probable pathway^29^. TPE, in particular, is utilized in designing mechanoluminescent materials, due to its capability of self-organisation and the ability to be incorporated into larger multicomponent assemblies *via* solvophobic control^30^. TPE lumiophores also widely used in various fields such as organic, biological, supramolecular, organic electronics and medicinal chemistry^31^. TPE applications include chemical sensors, optoelectronic devices, organic solar cells, light emitting diodes and field effect transistors^32^,^33^,^34^,^35^,^36^. There has been various chemical, physical, and molecular design approaches used to derivatise TPE luminophore, whose absorption and emission properties can be tuned to optimize their solubility in polar and non-polar solvents. For example, the attachment of bulky alicyclics, encapsulation in amphiphilic molecules, and blending with polymers etc.^31^. Although TPE is widely used in various fields, however, designing small organic molecules with the potential of producing well-organised supramolecular nanostructures with efficient luminescence in solid states is still rare.

## Results

**Figure 1:**
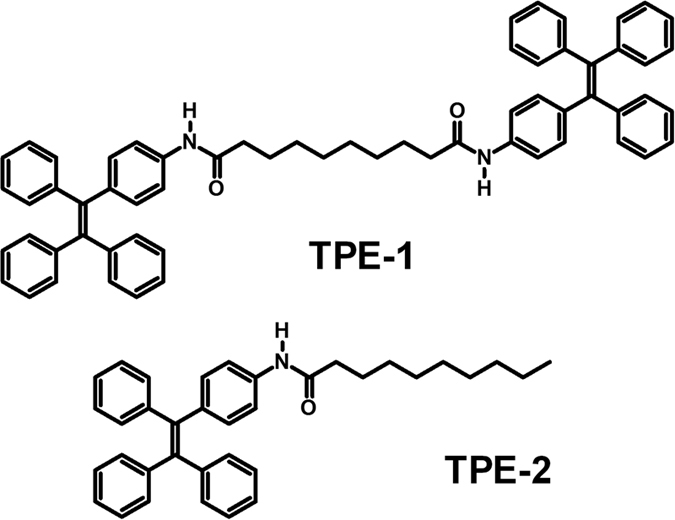
The chemical structure: dumbbell shaped TPE-1 and its amphiphilic analogue TPE-2 molecules used in this study.

We recently became interested in the design and development of novel AIE-active TPE luminogens with supramolecular self-assembly potential to produce variety of nanostructures such as star shaped^37^, controlled helical^38^, highly efficient near-IR solid emitter^39^, metal organic framework^40^, and use in various applications such as sensing of organic volatiles^41^, pH sensor^42^, bioimaging^43^, and donor-acceptor systems for solar cells^44^. As part of our on-going program, we investigated the synthesis of dumbbell-shape **TPE-1** and **TPE-2** amphiphilic molecules (Fig. 1).

Both **TPE-1** and **TPE-2** compounds were synthesized by amide coupling of amino-TPE and alkyl-dicarboxylic acid and alkyl-carboxylic acid in the presence of 1-ethyl-3-(3-dimethylaminopropyl)carbodiimide (EDCI) and 4-dimethylaminopyridine (DMAP) in dry DMF, the synthetic procedure shown in Scheme 1 (for details see ESI). Both compounds were characterized using ^1^H, ^13^C NMR, HRMS and elemental analysis. **TPE-1** and **TPE-2** are highly soluble in common organic solvents such as THF, chloroform, dichloromethane (DCM) and insoluble in acetonitrile, methanol, water and hexane.

**Figure 2:**
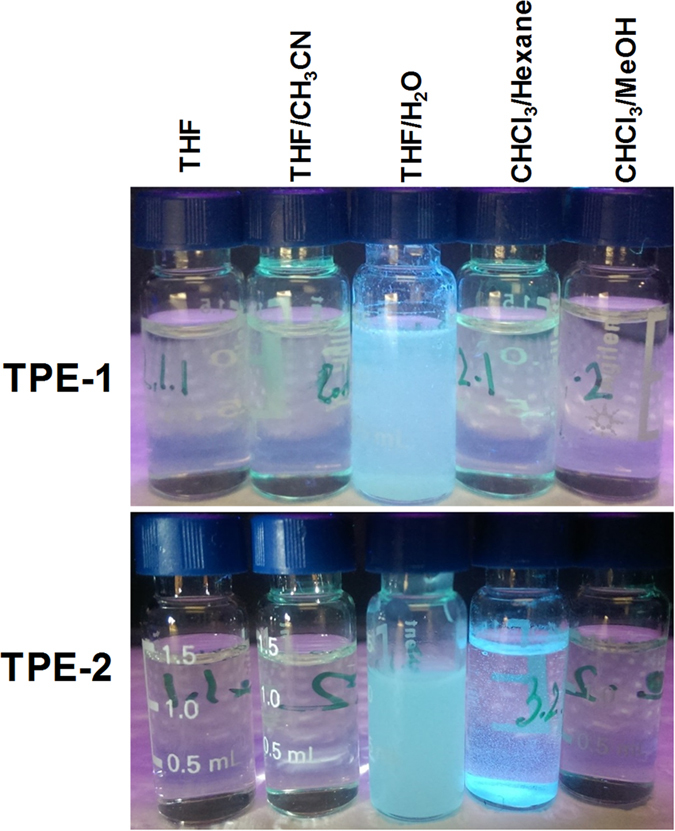
Luminescence properties: photographic images of TPE-1 and TPE-2 solutions in various solvent mixtures irradiated by UV light (λ_ex_ = 365 nm).

Figure 2 shows luminescence properties of **TPE-1** and **TPE-2** through solvophobic control. Compound **TPE-1** produces a faint emission in solution, while produces strong fluorescence in its aggregated state in THF/water and CHCl_3_/hexane, however, no FL was observed in THF/MeOH and THF/ACN. Interestingly, **TPE-2** only emits strong fluorescence in the aggregate state in THF/water. Here we use TPE as an extended π-conjugated moiety, amide group for a hydrogen-bonding and the alkyl chain as a segment that can introduce van der Waals interaction to the self-assembly process.

### UV-vis absorption and fluorescence spectroscopy

**Figure 3:**
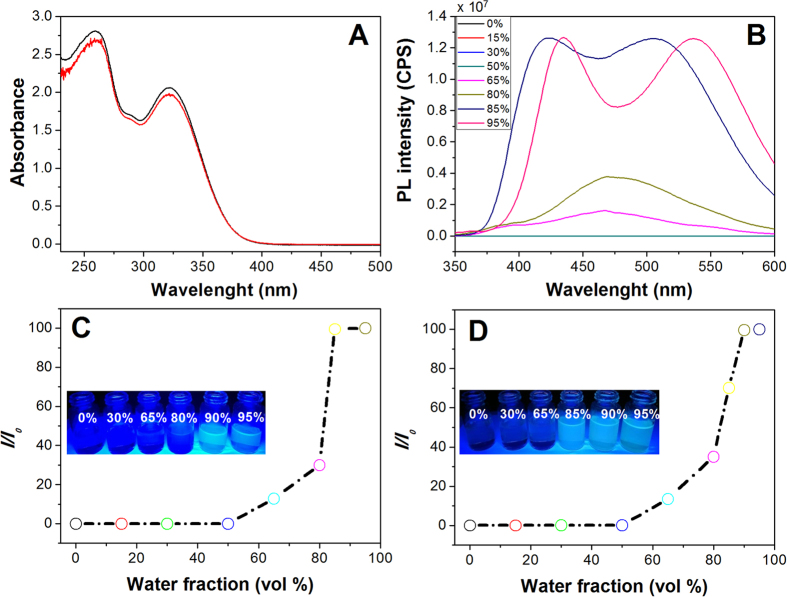
Solution based self-assembly. (**A**) The UV-vis absorption spectra of **TPE-1** and **TPE-2** (10 μM), (**B**) the fluorescence spectra of **TPE-1** (10 μM) in THF/water at various water fractions, (**C** and **D**) florescence intensity changes of **TPE-2** and **TPE-1** at 475 nm as a function of water fraction in THF solvent mixture and irradiation at 365 nm, respectively.

The photophysical properties of **TPE-1** and **TPE-2** were screened using UV-vis absorption and fluorescence spectroscopy in polar and mixture of polar and non-polar solvents as shown in Fig. 3. The UV–vis absorption spectrum of both **TPE-1** and **TPE-2** derivatives in THF (1 × 10^−5^ M) exhibited π-π* transition with absorption maxima at 325 and 260 nm (Fig. 3a). The photoluminescent (PL) spectrum of **TPE-1** in THF shows no detectable signals (Fig. 3b,c). Furthermore, the solvatochromic properties of **TPE-1** and **TPE-2** were surveyed at various water fractions (*f*_w_). No spectral changes were observed upon the increase of water fractions between 10 and 50% *v* in THF/water solvent mixture. Both luminogens become emissive when water fraction increases to *f*_w_ = 65–80% *v*, where PL spectra show a red-shifted of 15 nm and an enhanced emission of 25-fold at 475 nm due to molecular self-assembly. This is a clear verification of the AIE characteristics of **TPE-1** aggregates, owing to decreased solubility with increasing *f*_w_ in THF. Interestingly, at addition of 90% water fractions, the PL intensity enhanced rapidly, and the luminescence maxima split to two peaks one blue-shifted to 418 nm and the other red-shifted to 505 nm. Further increase of *f*_w_ to 95% *v* shifted the two luminescence maxima to 435 and 535 nm, respectively. At *f*_w_ = 95% *v*, a 70-fold enhancement of emission has been observed as compared to that in 100% THF. Similar phenomenon was also observed for **TPE-2** as shown in Figs 3d and S1. The fluorescence quantum yield (Φ_*F*_) of **TPE-1** and **TPE-2** in 100% THF solutions are ~0.05 and 0.09% which were enhanced by 70-fold to 3.61 and 6.78% at *f*_w_ = 95% *v* respectively, as measured with Rhodamine B as a standard with Φ_*F*_ = 70% in ethanol.

### Density functional theory

**Figure 4:**
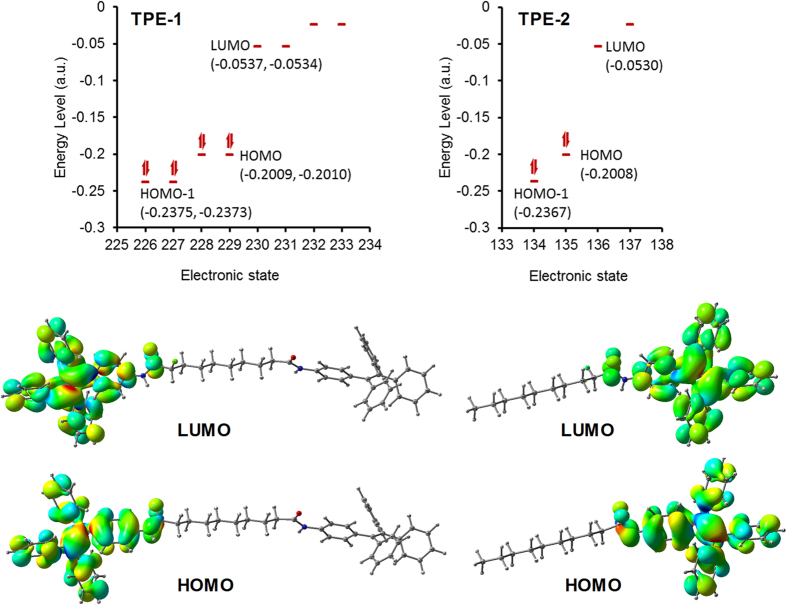
Density functional theory (DFT) calculations: Electron density distribution of HOMO and LUMO orbitals of TPE-1 and TPE-2.

Density functional theory (DFT) calculations with Gaussian 09 suite of programs and B3LYP/6-311 G level of theory^45^ was used to optimize molecular conformation of **TPE-1** and **TPE-2** in the gas phase and calculate the HOMO–LUMO gap. The similarity of the spectroscopic properties of **TPE-1** and **TPE-2** is due to the similarity of the electronic structure of these two compounds. The symmetry in **TPE-1** results in the degeneracy of the electronic states producing two states per energy level. The HOMO–LUMO gap of **TPE**-**1** and **2** are 309.6 nm (4.005 eV) and 308.4 nm (4.020 eV) respectively. Similarly, the HOMO-1 to LUMO transition for **TPE-1** and **TPE-2** required 248.2 nm (4.995 eV) and 248.1 nm (4.997 eV) wavelength respectively. These are very close values due to the electronic structure of chromophores in these two molecules. The distribution of electron density of the HOMO and LUMO orbitals of **TPE-1** and **TPE-2** show similar distributions as shown in Fig. 4.

### Field Emission Scanning Electron Microscopy

**Figure 5:**
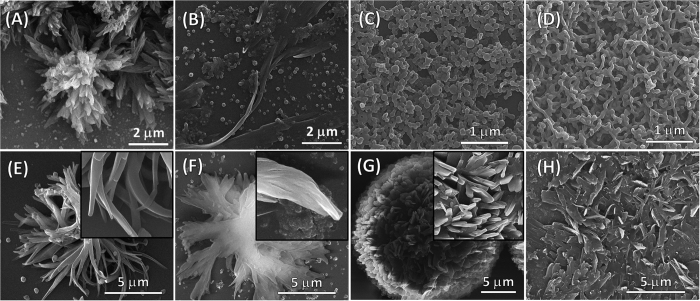
Visualisation of various self-assemblies by SEM analysis. SEM micrographs of microstructures of **TPE-1** from solutions in (**A**) *f*_w_ = 70%, (**B**) *f*_w_ = 80%, (**C**) *f*_w_ = 85%, (**D**) *f*_w_ = 95%, and **TPE-2** deposited from solutions in (**E**) *f*_w_ = 70%, (**F**) *f*_w_ = 80%, (**G**) *f*_w_ = 85%, (**H**) *f*_w_ = 95% of water-THF solvent mixtures.

The dumbbell shaped **TPE-1** produced nano-sphere particle about 80–120 nm in diameter when deposited from a solution with *f*_*w*_ = 85% in water-THF solvent mixture (Fig. 5C). These particles tend to aggregate at *f*_*w*_ = 95% forming a network of fused particles (Fig. 5D). These particles nucleate in the solution due to lower solubility of **TPE-1** in high *f*_*w*_, with no surface initiated self-assembly occurs during solvent evaporation on the substrate at these high *f*_*w*_. At lower *f*_*w*_ of 80% we observe an increased **TPE-1** solubility of and thus a combination of nanoparticles and ribbons and ribbon aggregates, which form upon solvent evaporation (Fig. 5B). The solubility of **TPE-1** increases further at *f*_*w*_ = 70%, the self-assemblies formed from this solution are flower like fractal microstructures composed of short ribbons grown radially from one centre on the substrate surface. This can be justified by the coexistence of oligomer aggregates and molecular **TPE-1** in solution, where the solubilized molecules produce surface initiated self-assemblies during solvent evaporation (Fig. 5A).

**TPE-2** produced aggregated ribbons at *f*_w_ = 70 and 80% (Fig. 5E,F). Fractal flowerlike structures were produced at *f*_w_ = 85% (Fig. 5G). These microstructures are single centred, half spherical, fractals composed of crystalline nanosheets about 120 nm in thickness and 0.5–1.0 μm in width (Fig. 5G inset). This fractal structure initially grows radially on the substrate surface, and at later growth stages fill a 3D half sphere, giving a fractal flower-like structure. Increasing water fractions to 95%, **TPE-2** produced irregular sheets deposition (Fig. 5H).

The **TPE-2** fractal self-assemblies showed a defined crystallinity as evidenced by the defined X-ray diffraction (XRD) pattern (See Figure S2)^26^. This crystallinity arises from the ribbons composing the fractal structures, which may be induced by the crystallinity of silicon wafer surface, directing the self-assembly of the initial layer in a preferred orientation resulting in crystalline growth in a specific plane.

**Figure 6:**
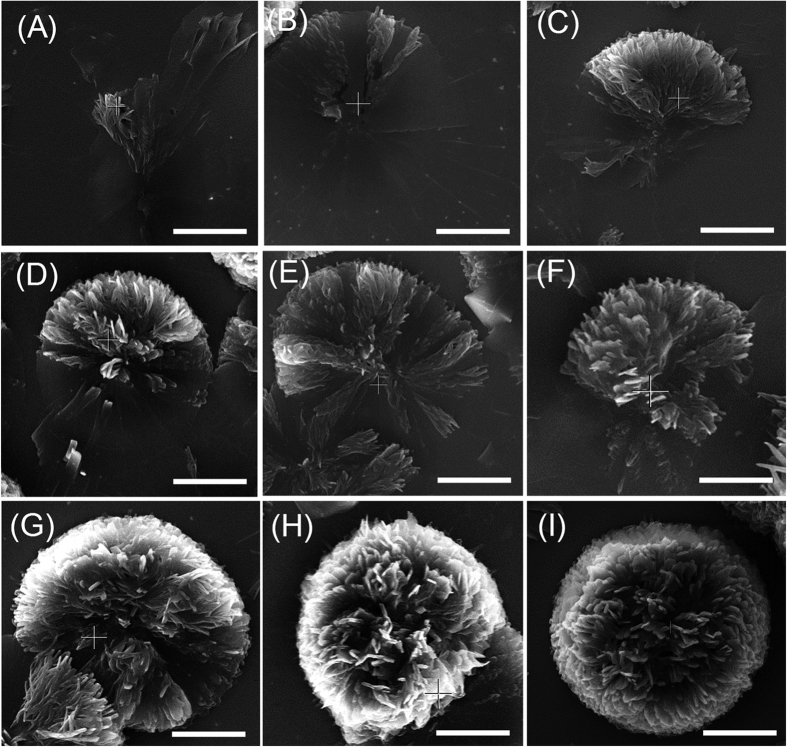
Flower like assembly formation process. SEM micrographs of **TPE-2** deposited by solvent evaporation of water-THF (*f*_w_ = 85%) solutions, showing a step-by-step growth of the flower-like 3D fractal microstructure (The scale bar indicates 10 μm).

Direct visualisation of the Self-assembled microstructures using SEM provides unique insight in the mechanism of flower assembly. Figure 6 clearly shows the step-wise growth mechanism of 3D flower like fractals of **TPE-2** in water-THF (*f*_w_ = 85%). The samples were prepared by solvent evaporation over 3 hours and the SEM images taken were of formations at various stages of growth of the same sample. The similarity in the self-assembly process of **TPE-1** and **TPE-2** compounds is because **TPE-2** can initially self-assemble into a dimer which is structurally similar to **TPE-1**. The higher solubility of the **TPE-2** due its lower molecular weight, allows the formation of flower-like microstructures from higher *f*_w_. The flower like structure is a 3D hierarchically assembled supramolecular fractal architecture formed from non-planar two dimensional sheets, which can collapse to form crystalline solid (see Supplementary Information Figure S5). The sheets grew randomly in less populous regions of the substrate at higher *f*_w_ (Fig. 5H).

Furthermore, optical microscopy clearly support formation of flower structures by solvent evaporation of water-THF (*f*_w_ = 85%) solutions at room temperature (Figure S7). However, failed to obtained *in-situ* growth of flower by optical microscopy it may be due to decomposition of assembly by heating under flashlight from the microscopy when taking the image (Figure S8). Transmission electron microscopy (TEM) clearly shows fractal of flower microstructure on carbon grid (Figure S9d).

### Mechanochromic properties

**Figure 7:**
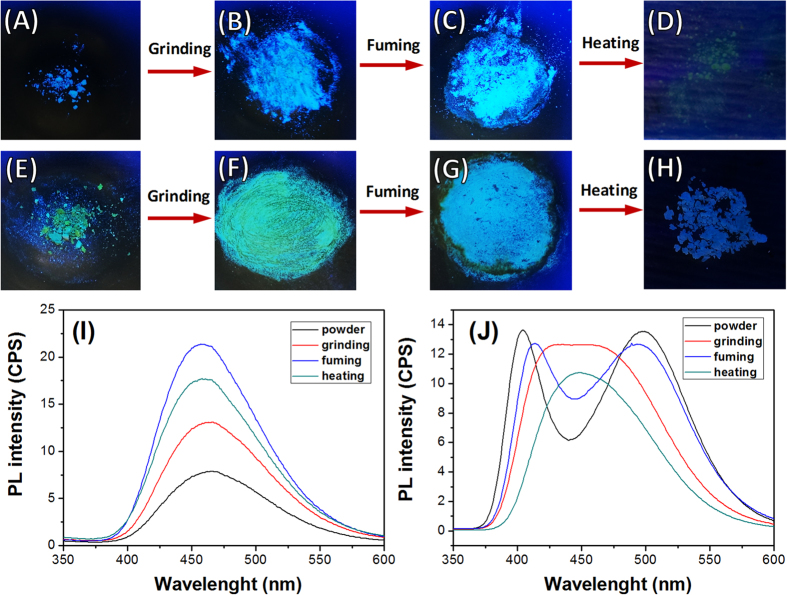
The mechanochromic properties. **TPE-1** (**A**–**D**) and **TPE-2** (**E**–**H**) showing the luminescence of these two compounds after grinding, fuming and heating. The florescence spectra of (**I**) **TPE-1**, and (**J**) **TPE-2** at the powder crystalline, ground, fumed and heated states, respectively.

Both **TPE-1** and **TPE-2** show mechanochromic properties which can be observed in the process of grinding, fuming and heating, respectively as shown in Fig. 7. The luminescence properties of such compounds are subject to the molecular packing in the solid state. This is in agreement with AIE behaviour of **TPE-1** and **TPE-2** in solution, where the stacking of the TPE moieties in self-assembly hinders non-radiative relaxation pathways, the same can occur in the crystalline solid state. Grinding **TPE-1** and **TPE-2** gave powders with the same emission colour, which is an evidence of retaining the crystalline structure in the process. The reduction in the intensity of the ground material is due to the reduction in the crystallite size distribution. Fuming the ground materials reverted them to their initial luminescent states in colour and intensity. Heating the grounds at 100 °C for 3 min has reduced the luminescence in both **TPE-1** and **TPE-2** (Fig. 7D,H).

The solid state florescence spectra of **TPE-1** and **TPE-2** at various stages show a similar trend to the spectra at various *f*_w_ in solution, where various aggregate sizes are produced (Fig. 7I,J). **TPE-1** initial powder, as shown in the SEM images, is composed of nanospheres with very fine particle size, and therefore giving weak luminescence at 477 nm. Grinding this material fused some particles increasing the particle size distribution and enhanced the florescence intensity. Furthermore, fuming the ground material increased crystallites size and further enhanced luminescence intensity with a blue shift to 470 nm. Heating the fumed material gives an amorphous material with less restriction on the non-radiative relaxation pathway and resulting in significant decrease in luminescence intensity. As we have seen in solution, **TPE-2** shows a different luminescence behaviour in comparison to **TPE-1**. The initial powder of **TPE-2** is well crystalline material which gives two peaks at 407 and 498 nm. Grinding this material changes the florescence pattern to a single broad peak around 450 nm. After fuming the ground **TPE-2**, the spectrum reverted to two peaks at 425 and 485 nm with a red shift in the first and blue shift in the second bands. Heating the fumed powder reduces luminescence and reverts the luminescence spectrum of two peaks pattern to a single peak at 448 nm. This shows the strong sensitivity of **TPE-1** and **TPE-2** molecular stacking in the crystal structure to thermal and mechanical forces, where heating can allow the non-radiative relaxation pathways due to formation of amorphous material with reduced luminescence.

**Figure 8:**
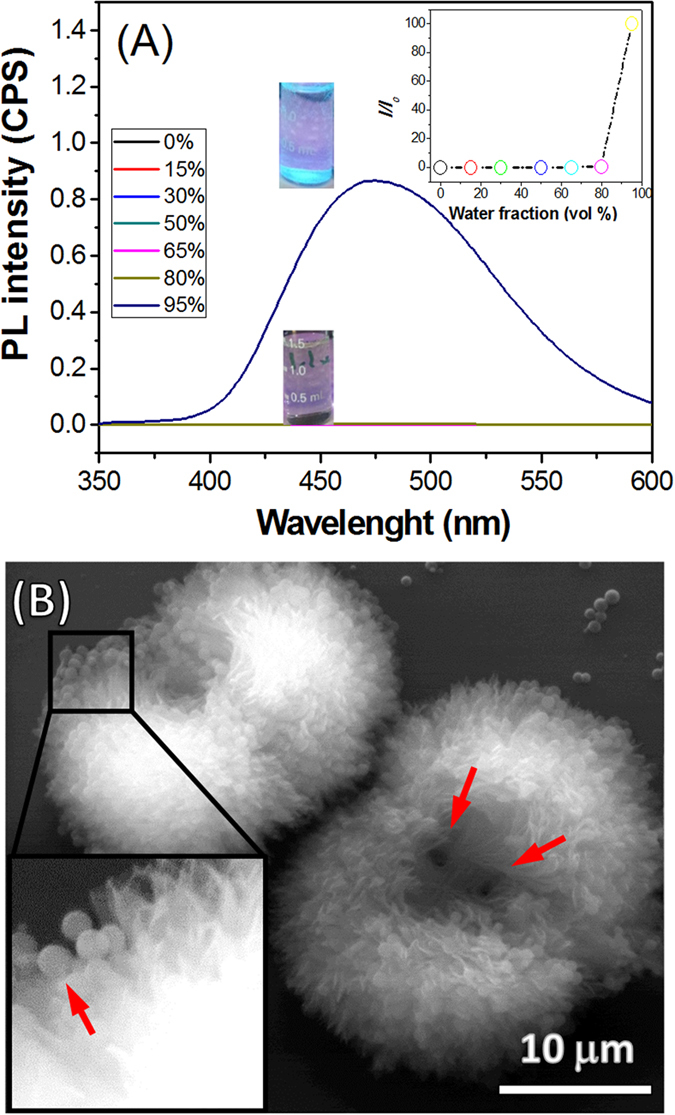
(**A**) The fluorescence spectra of **TPE-1** in CHCl_3_/hexane solvent mixtures, and (**B**) SEM micrograph of the fractal microstructure of **TPE-1** deposited from CHCl_3_/hexane at *f*_h_ = 95% v on a silicon wafer substrate, the inset shows a magnified area of the image where nanospheres can be seen.

The AIE behaviour of **TPE-1** in chloroform-hexane solvent mixtures is similar to trend seen for this compound in THF-water (Fig. 8A). The better solubility of **TPE-1** in organic solvents in comparison to THF-water has shifted the AIE in solution to high ratios of hexane ~95% *v*. The self-assembly of **TPE-1** when deposited from *f*_h_ = 95% *v* in chloroform-hexane on a silicon wafer, shows flower like microstructure as well as nanoparticles. The two centres of the fractal are positioned between two lobes and 4–5 μm apart (Fig. 8B). The flower like microstructures are two lobed with dual centred fractal growth and 20–35 μm in diameter, composed of sheets few tens of nanometers in thickness.

**Figure 9:**
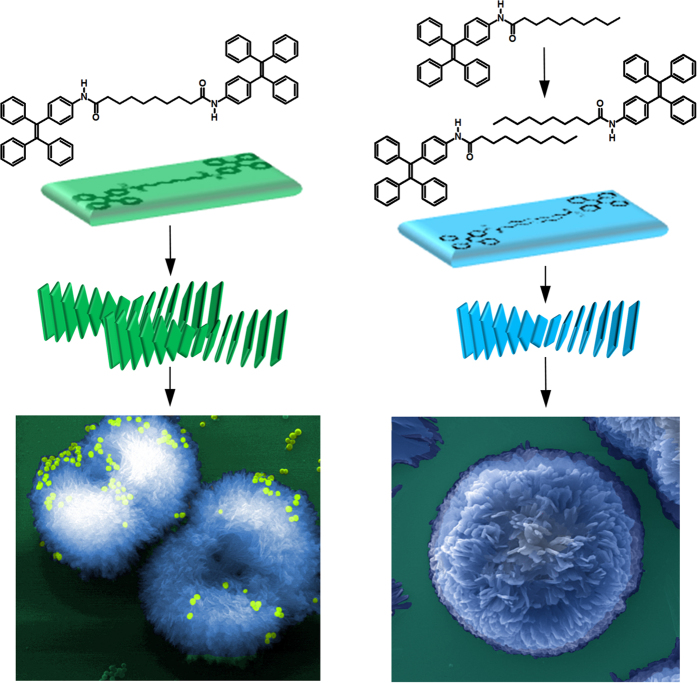
Schematic diagram illustrating flower-like assembly of TPE-1 in CHCl_3_/hexane (*f*_h_ = 95%) and TPE-2 in THF/water (*f*_w_ = 85%). False coloured SEM images used to visualise the nanospheres and flower-shape assembly.

This work demonstrates that complex self-assembly can indeed be attained through hierarchical non-covalent interactions i.e. hydrogen-bonding, π-π interaction and van der Waals forces through solvophobic control. Figure 9 illustrates a schematic arrangement of **TPE-1** CHCl_3_/hexane (*f*_h_ = 95%) and **TPE-2** in THF/water (*f*_w_ = 85%) to form stacks which can further hierarchically assemble to form the larger flower like structures due to the solvophobic interactions and H-bonding.

## Discussion

The formation of micrometer-sized flower-like supramolecular structures was obtained by hierarchical self-assembly of a tetraphenylethene (TPE) derivatives in aqueous–organic solvent mixture medium for the first time. It can be noted that the flower-like microsctructures several micrometers in size (10–30 μm) are composed of flake-like nanostructures several nanometers in thickness. The solution of **TPE-1** in CHCl_3_/hexane (*f*_h_ = 95%) and **TPE-2** in THF/water (*f*_w_ = 85%) were aged for at least three hours and then evaporated to allow for subsequently air drying on a silicon wafer substrate, this has left flower-like superstructure on the substrate surface. The formation of such flower-like fractal assembly is attributed to a balance between the intermolecular interactions, including the π-π interaction that require a perfect stacking alignment and H-bonding and van der Waals interactions that require a torsion adjustment angle to relieve the hindrance in the self-assembly which results in a smaller intermolecular torsion angle. This allows molecules to form supramolecular assemblies in a controlled 3D fashion as illustrated in Fig. 9. Similar to many other TPE derivatives, **TPE-1** and **TPE-2** show AIE behavior in aggregated state that can form in a bad solvent media such as high water fraction mixtures with organic solvents. Interestingly, **TPE-2** in THF/water (*f*_w_ = 85%) self-assembled into flower-like structures. However, **TPE-1** assembled into spheres in similar water contents and dual centred two lobe surface initiated fractal growth in CHCl_3_/hexane upon solvent evaporation.

The SEM analysis importantly shows the ability to build microstructures based on self-organization using solvophobic effect and molecular noncovalent interactions into well-defined and discrete complex morphologies such as flower-like objects. This may inspire further advances molecular superstructural design based on self-assembly theory that go beyond simple morphologies. To the best of our knowledge, the flower-shaped morphology forming from an AIE-active molecule in the aqueous medium described herein is the first examples of such assembly being observed in a supramolecular system containing single molecule. The results described in this paper demonstrate an actionable roadmap to about how to handle intermolecular interaction in molecular design and utilizing the solvophobic effects and thermal and mechanical stimuli which can guide further advances in supramolecular geometrical design and functionality. Furthermore, flower-like structures of AIE-active molecule built using solvophobic control indicate their potential application in various fields in combination with other chemical entities.

## Materials and Methods

TPE, chloroform (CHCl_3_), chloroform-d (CDCl_3_), methanol (MeOH), dichloromethane (DCM), Tetrahydrofuran (THF), *N,N*′-dimethylformamide (DMF) were purchased from Aldrich and used without purification, unless otherwise specified. Fluorescence measurements were performed on a FluoroMax-4, Horiba Jobin Yvon, equipped with an injector port, a stirrer and a temperature controller (25 °C). ^1^H NMR, ^13^C-NMR spectra were recorded on a Bruker spectrometer using CDCl_3_ and MeOD as solvent and tetramethylsilane as an internal standard. The solvents for spectroscopic studies were of spectroscopic grade and used as received. Mass spectra (MS) were obtained by using Bruker AutoFlex Matrix Assisted Laser Desorption/Ionisation (MALDI) Time of Flight (TOF)-Mass Spectrometer (MALDI-TOF-MS). The X-ray diffraction (XRD) pattern spectra were performed on a Bruker D8 FOCUS diffractometer using a Cu target radiation source (λ = 0.15418 nm).

### Spectroscopic measurements

#### UV–Vis measurements

UV–Vis absorption spectra were recorded in a Cary-50, and UV–Vis spectrometer in 1 cm path length cuvette. A 0.2 mL aliquot of the stock solution of **TPE** (conc. = 10^−3^ M) was transferred to various ratios ACN/THF in different volumetric flasks, and made up to 2 mL volume. The solutions were allowed to equilibrate for 2 h prior to the spectroscopic measurements.

#### Fluorescence Measurements

Fluorescence emission spectra were recorded in a Horiba Jobin Yvon FluoroMax^®^-4–Spectrofluorometer. Fluorescence measurements and quenching experiments were performed on a FluoroMax-4 equipped with an injector port and stirrer at 25 °C. All experiments were performed in a quartz cell with a 1 cm path length with 365 nm excitation wavelength.

#### SEM imagining

The silicon wafer was cleaned by acetone, ethanol and then Milli Q water. SEM Samples were prepared by solvent evaporation on a silicon wafer and then sputter coated with gold for 10 s at 0.016 mA Ar plasma (SPI, West Chester, USA) for SEM imaging using a FEI Nova NanoSEM (Hillsboro, USA) operating at high vacuum which provided direct visualisation of the self-assembled aggregated structures.

#### Transmission Electron Microscopy (TEM) imagining

TEM samples were prepared by solvent evaporation on a holey carbon grid and micrographs were produced using a Jole 1010 100 kV TEM.

#### Fourier Transform Infrared Spectroscopy (FTIR)

FTIR spectra were collected on a Perkin Elmer FT-IR 400 at ambient temperature. The instrument was continuously purged with CO_2_-free dry air.

#### Quantum efficiency (Φ_*F*_)

The fluorescence quantum efficiency (Φ_*F*_) of the samples with absorption (intensity ~0.05) was estimated using fluorescein in ethanol (Φ_*F*_ = 70%) as standard solution and Φ_*F*_ of the solid films was measured using an integrating-sphere photometer.

#### Molecular modeling

Density functional theory (DFT) calculations with no consideration of dispersion interactions in gas phase were conducted using Gaussian 09 suite of programs.

## Additional Information

**How to cite this article:** Salimimarand, M. *et al*. Flower-like superstructures of AIE-active tetraphenylethylene through solvophobic controlled self-assembly. *Sci. Rep.* **7**, 42898; doi: 10.1038/srep42898 (2017).

**Publisher's note:** Springer Nature remains neutral with regard to jurisdictional claims in published maps and institutional affiliations.

## Supplementary Material

Supplementary Information
