# Supplementary material for: Flower-like superstructures of AIE-active tetraphenylethylene through solvophobic controlled self-assembly
Source: Sci Rep. 2017 Feb 23;7:42898. doi: 10.1038/srep42898 (PMC5322366; doi:10.1038/srep42898)

**Supplementary Information**

**Flower-like superstructures of AIE-active tetraphenylethylene through solvophobic controlled self-assembly**

Mina Salimimarand, Duong Duc La, Mohammad Al Kobaisi and Sheshanath V. Bhosale^*^

School of Science, RMIT University, GPO Box 2476, Melbourne, Vic. 3001, Australia

Corresponding authors: Tel.:+61399252680; E-mail: sheshanath.bhosale@rmit.edu.au

**
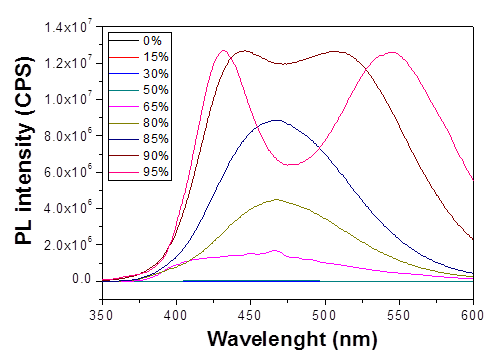
**

**Fig. S1**Fluorescence spectra of **TPE-2** (10 μM) in THF/water at various water fractions.

**Fig. S2** Powder XRD of **TPE-2** deposited in the f_w_ of 85% to produce flower like fractals (blue), 80% to produce nanosphere (black) self-assemblies and ground material (red).


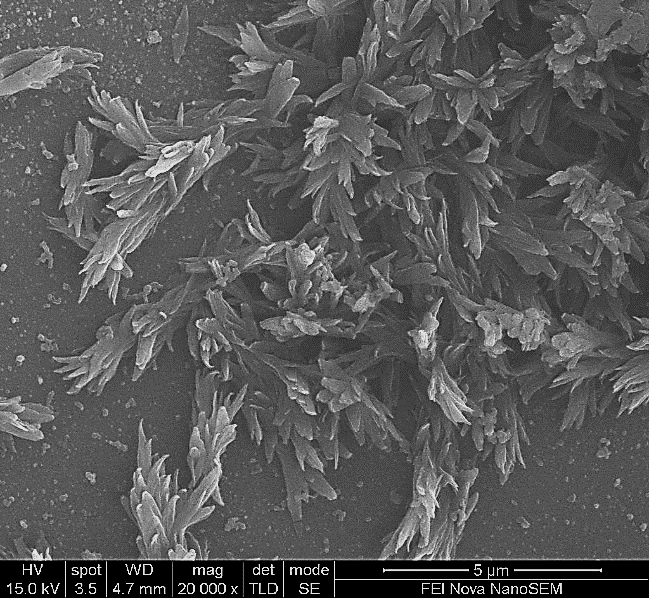

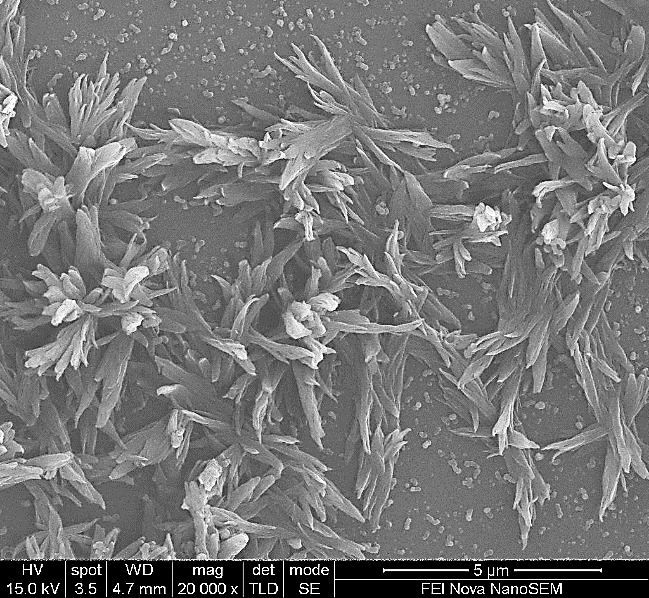


**Fig. S3** **TPE-2** deposited from f_w_ = 70% in THF-water mixture.


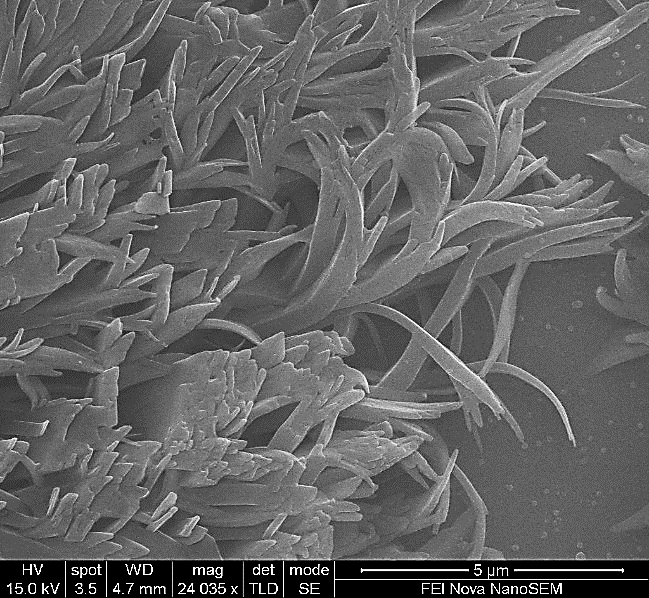

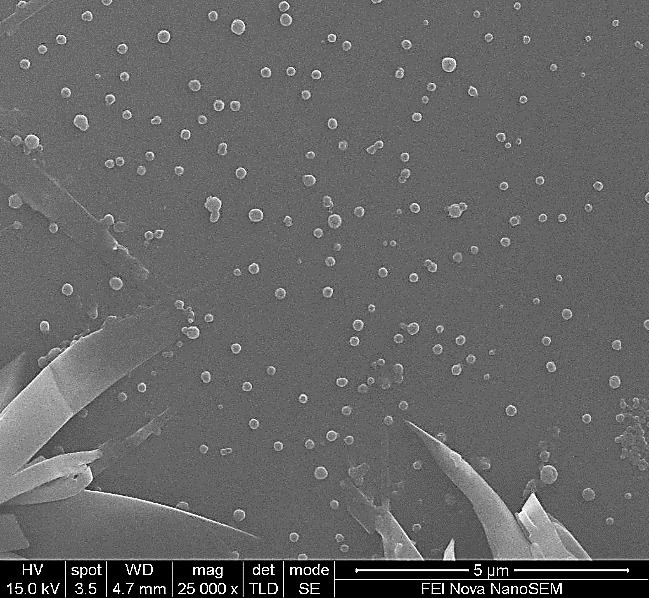


**Fig. S4** **TPE-2** deposited from f_w_ = 80% in THF-water mixture.


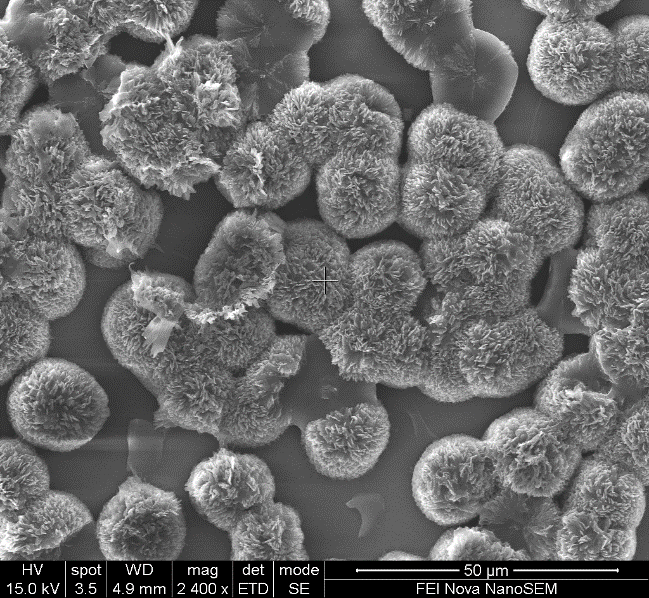

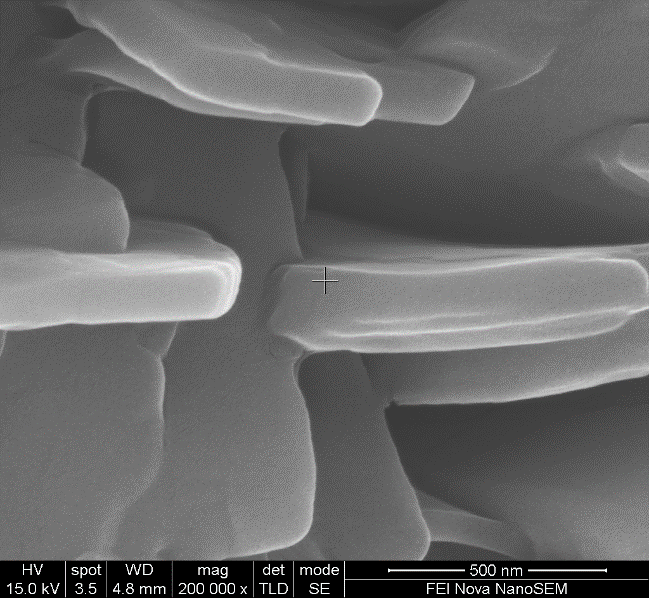


**Fig. S5 TPE-2** deposited from f_w_ = 85% in THF-water mixture.


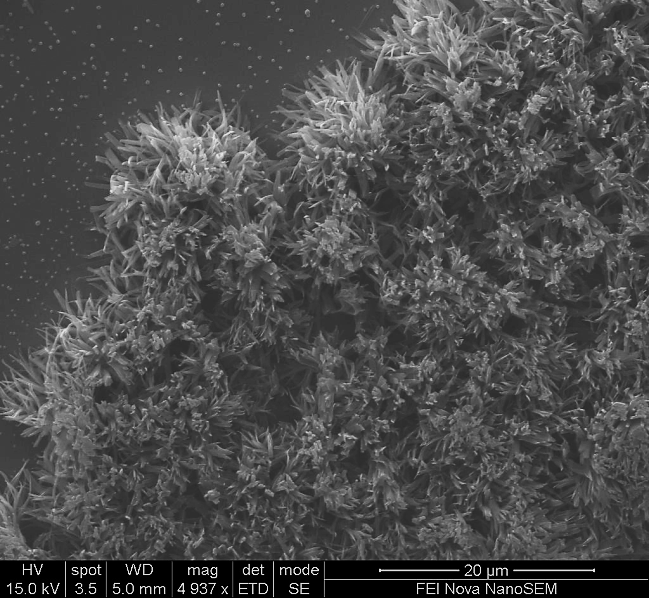

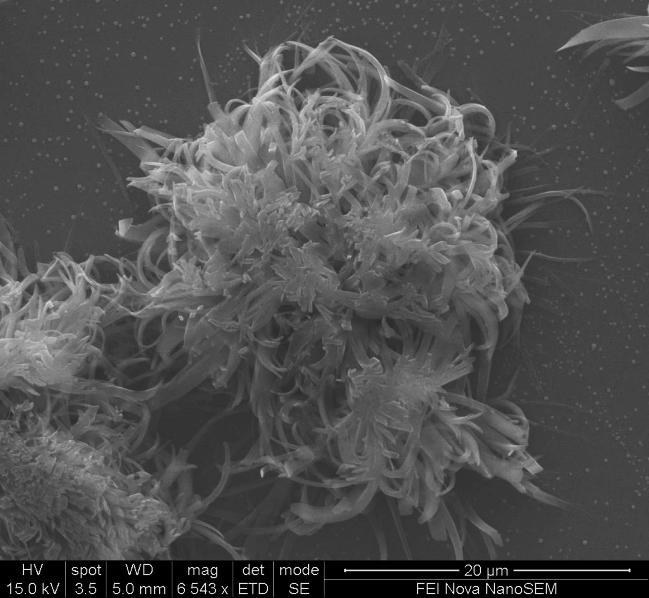


**Fig. S6 TPE-1** deposited from f_w_ = 70% in THF-water mixture.


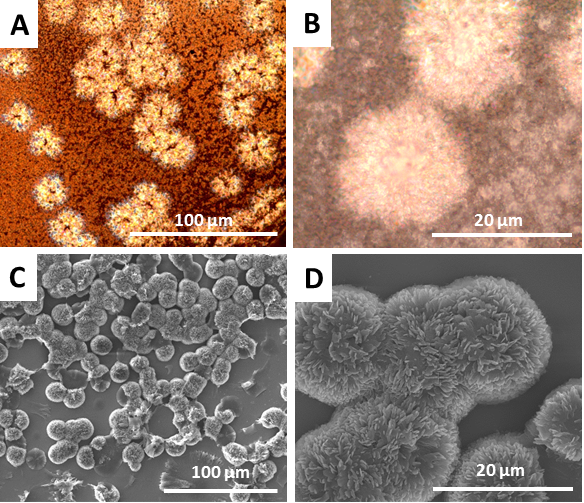


**Fig. S7** Optical images (Fig. A and B) and SEM images (Fig. C and D) of **TPE-2** deposited on silicon wafer by solvent evaporation of water-THF(f_w_ = 85%) solutions.


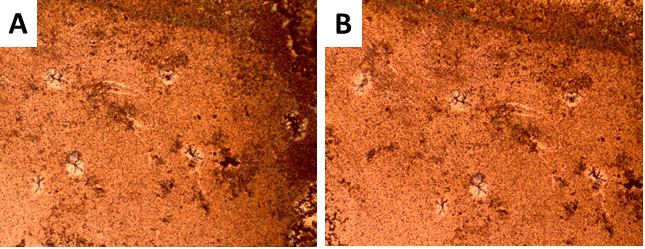


**Fig. S8** Optical images of **TPE-2** deposited on silicon wafer with water-THF (f_w_ = 85%) solutions after taking in-situ microscopy imaging.

**
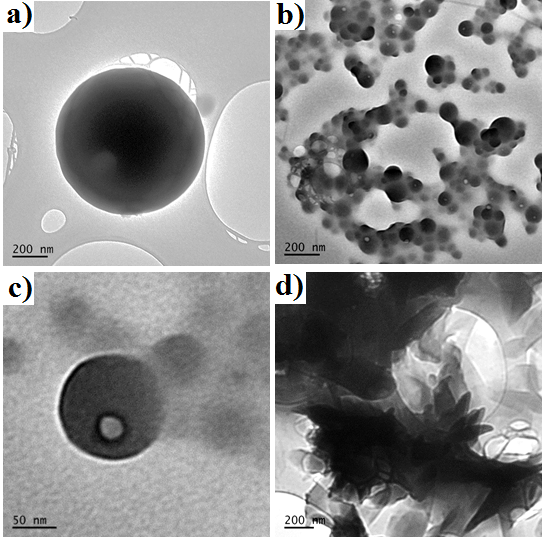
**

**Fig. S9** TEM micrographs of microstructures of **TPE-1** and **TPE-2**: a & c) **TPE-1** deposited from *f_w_* = 80 and 85% % from THF-water mixture. b) **TPE-1** from *f*_w_ = 95%, and d) **TPE-2** deposited from *f*_w_ = 85% in THF-water mixture.

**Synthesis of TPE derivatives**

**Scheme 1**. Synthesis of dumbbell shaped **TPE-1** and its analogue amphiphilic **TPE-2** molecules.

Synthesis of **TPE-1**: Sebacic acid (0.1 g, 0.5 mmol) and amino-TPE (0.407 g, 1.08 mmol) added to DMF (8 ml) at 0 °C and stirred for 10 min under nitrogen atmosphere, followed by addition of EDCI (0.230 g, 1.48 mmol), DMAP (0.181 g, 1.48 mmol) and TEA (0.2 ml, 1.40 mmol) at once. The reaction mixture stirred overnight at room temperature. Reaction completion was checked by TLC analysis. The reaction mixture was then diluted in chloroform followed by washing with 1M HCl then NaHCO3 10%, and water to remove excess DMF. The crude produced after evaporation was washed with methanol and 2-4 drops of HCl and was filtered to remove excess amine, the white-coloured solid of **TPE-1** was obtained (0.4 g, 43%). ^1^H NMR (300 MHz, CDCl_3_) δ 7.13-6.96 (m, 38H), 2.33-2.28 (m, 4H), 1.70-1.58 (m, 4H), 1.34-1.27 (m, 8H); ^13^C NMR (75 MHz, CDCl_3_) δ 171.1, 143.7, 143.6, 132, 131.4, 131.3, 127.8, 127.7, 127.6, 126.4, 126.3, 118.7, 77.4, 77, 76.6, 37.7, 29, 28.9, 25.4; Mass (ESI): m/z = HRMS m/z: calculated for C_62_H_56_N_2_O_2_ 861.1420, found 861.1427 (M)^+^ found; Elemental analysis for C_62_H_56_N_2_O_2_: calculated = C, 86.48; H, 6.56; N, 3.25; found = C, 86.53; H, 6.62; N, 2.87.

Synthesis of **TPE-2**: Decanoic acid (0.2 g, 1.2 mmol) and amino-TPE (0.243 g, 0.64 mmol) added to DMF (8 ml) at 0 °C and stirred for 10 min under nitrogen atmosphare, EDCI (0.218 g, 1.4 mmol) followed by DMAP (0.235 g, 1.92 mmol) and TEA (0.1 ml, 0.71 mmol) were added into the mixture. The reaction runs overnight in room temperature. Reaction completion was checked by TLC analysis. Crude was purified by column chromatography using Hexane and DCM to obtain **TPE-2** as a white colour solid (0.22 g, 64%). ^1^H NMR (300 MHz, CDCl3) δ 7.14-6.97 (m, 19H), 2.34-2.29 (m, 2H), 1.75-1.59 (m, 2H), 1.33-1.29 (m, 12H), 0.92-0.88 (m, 3H); ^13^C NMR (75 MHz, CDCl_3_) δ 171.1, 143.7, 143.6, 140.7, 140.3, 139.5, 136.2, 132, 131.4, 131.3, 127.8, 127.7, 127.6, 126.5, 126.4, 126.3, 118.7, 77.4, 77.2, 77, 76.6, 37.9, 31.8, 29.4, 29.3, 29.2, 25.6, 22.6, 14.4; HRMS m/z: calculated for C_36_H_39_NO 501.7140, found 501.7148 (M)^+^; Elemental analysis for C_36_H_39_NO: calculated= 86.18; H, 7.84; N, 2.79, found = C, 86.21; H, 7.91; N, 2.82,


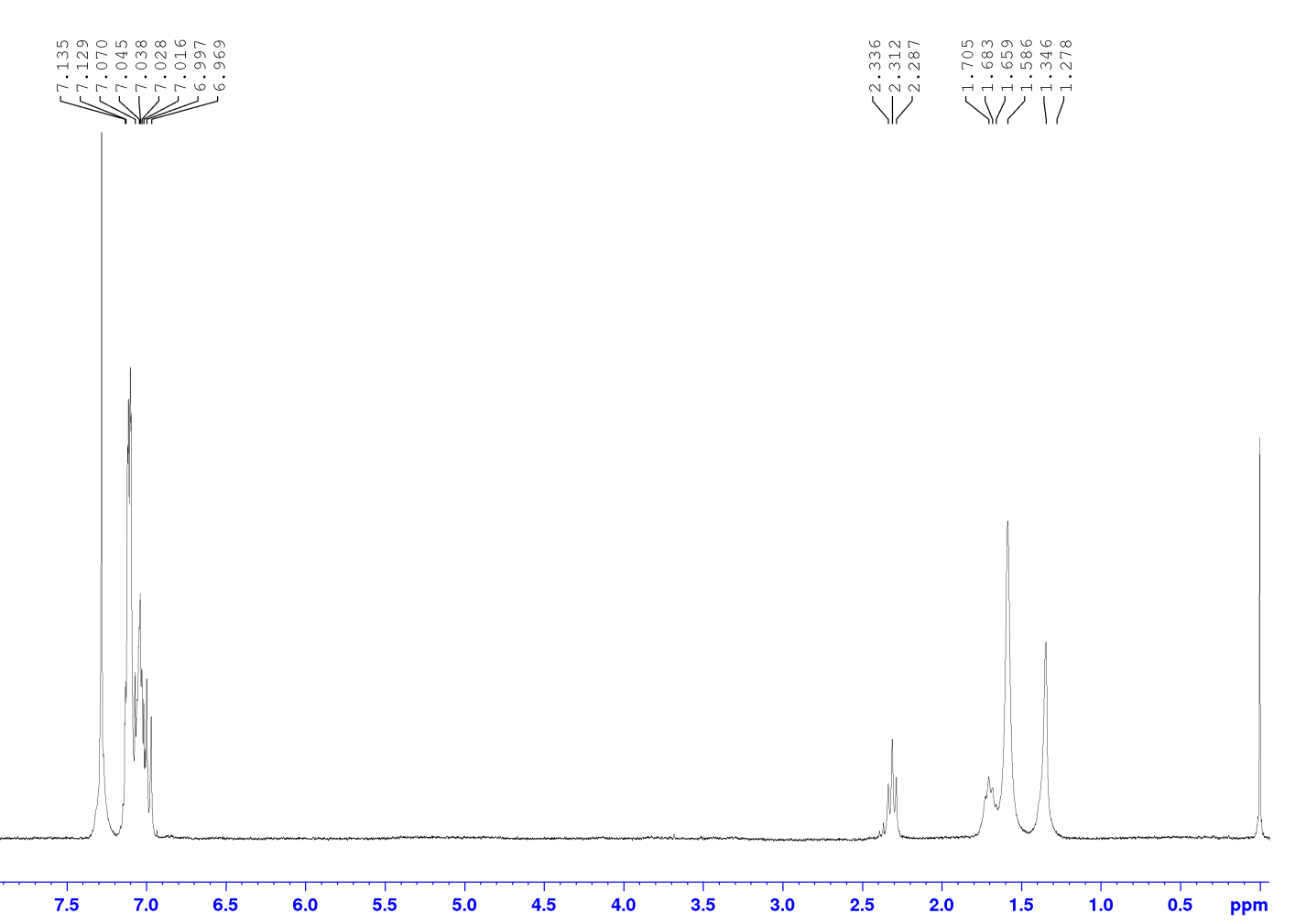


^1^H NMR of **TPE-1**


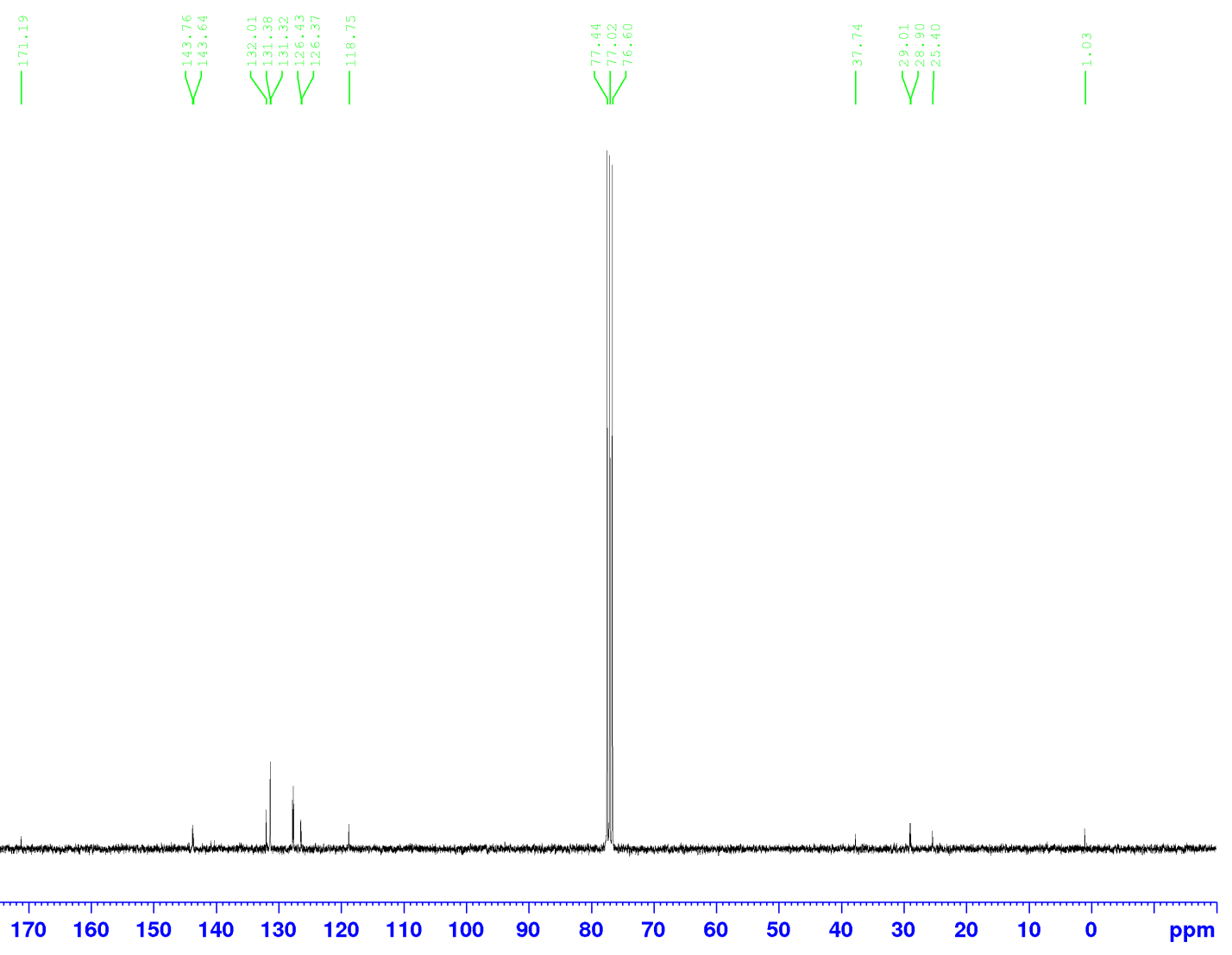


^13^CNMR of **TPE-1**


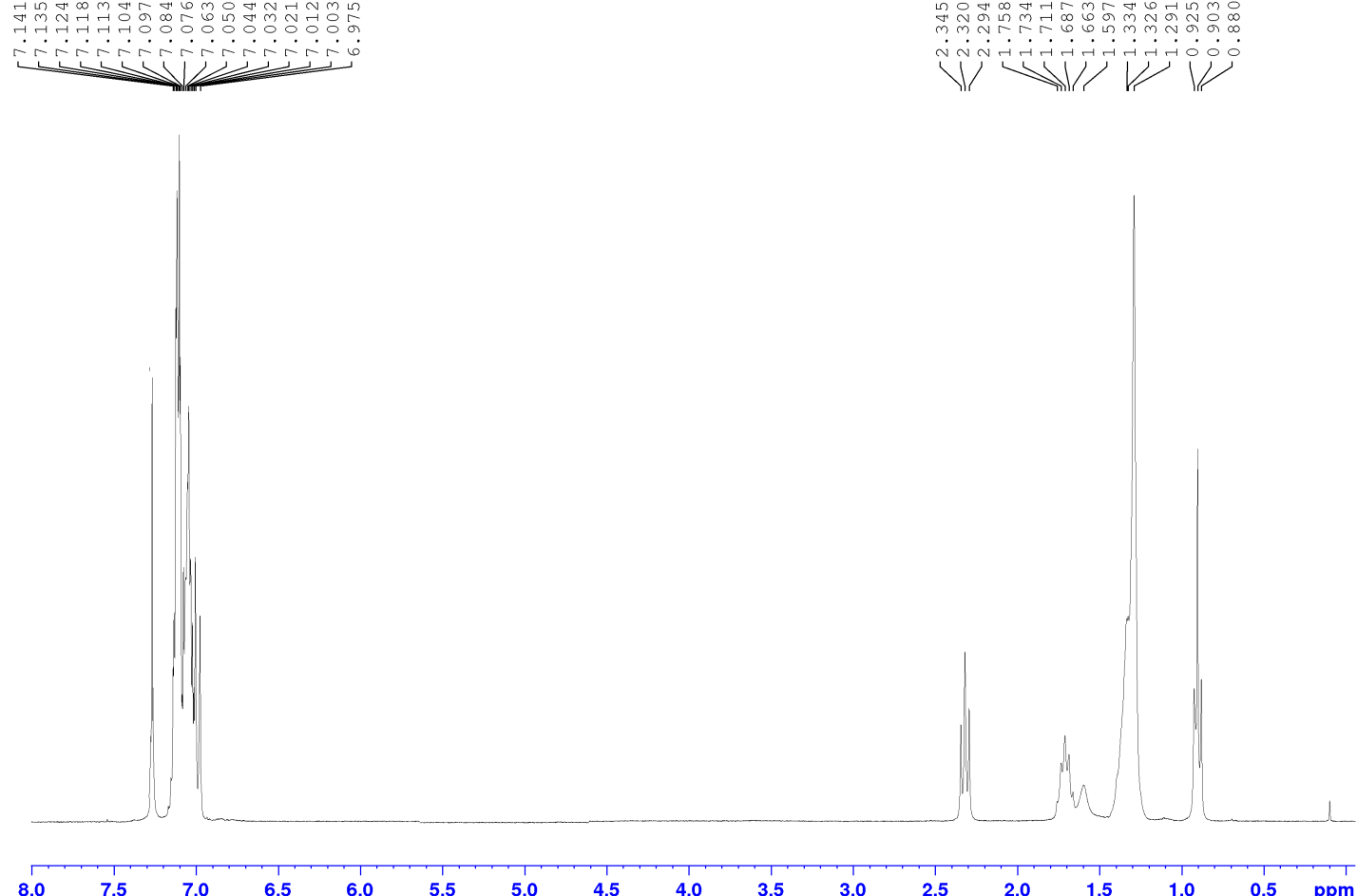


^1^H NMR of **TPE-2**


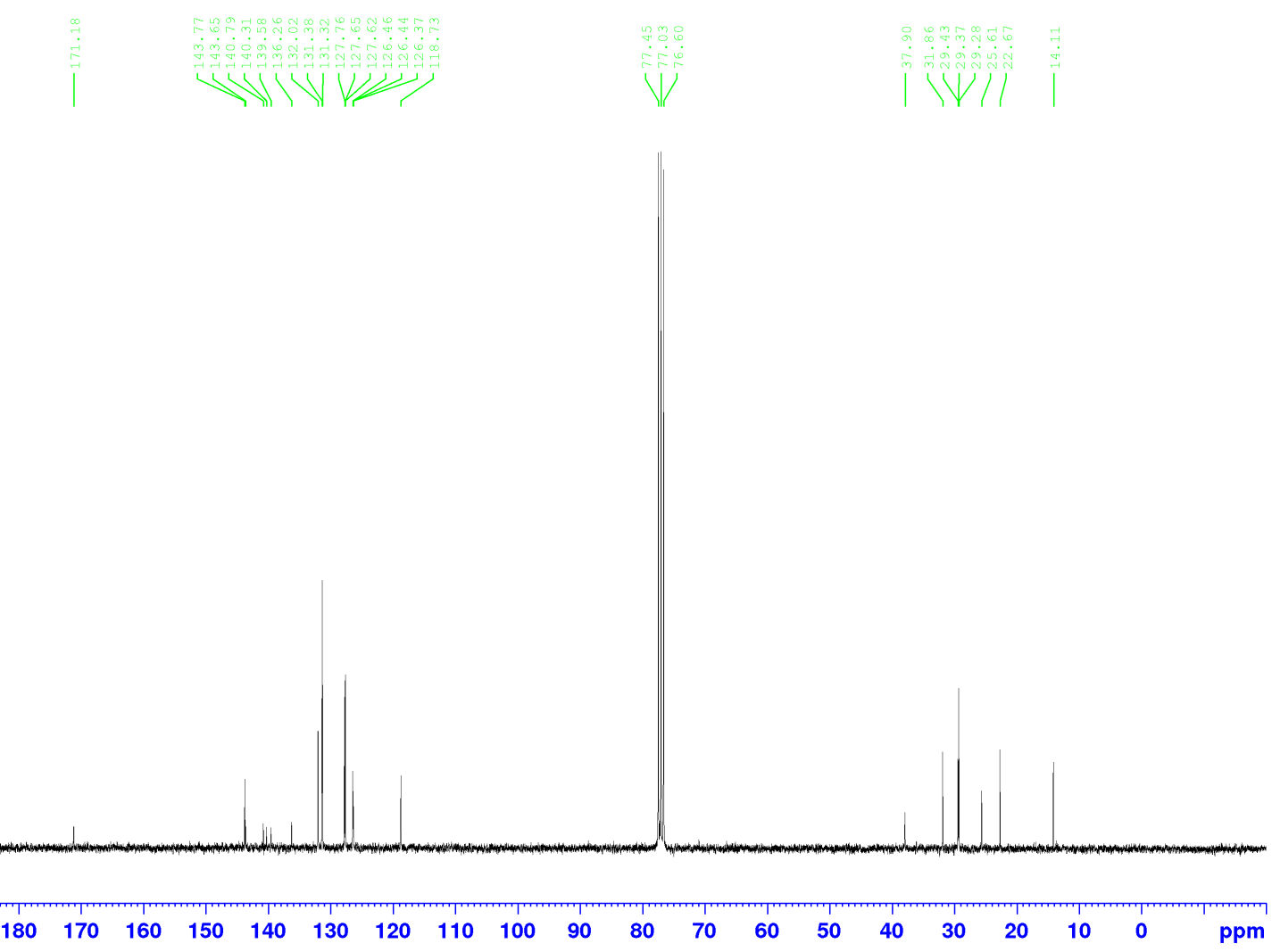


^13^C NMR of **TPE-2**


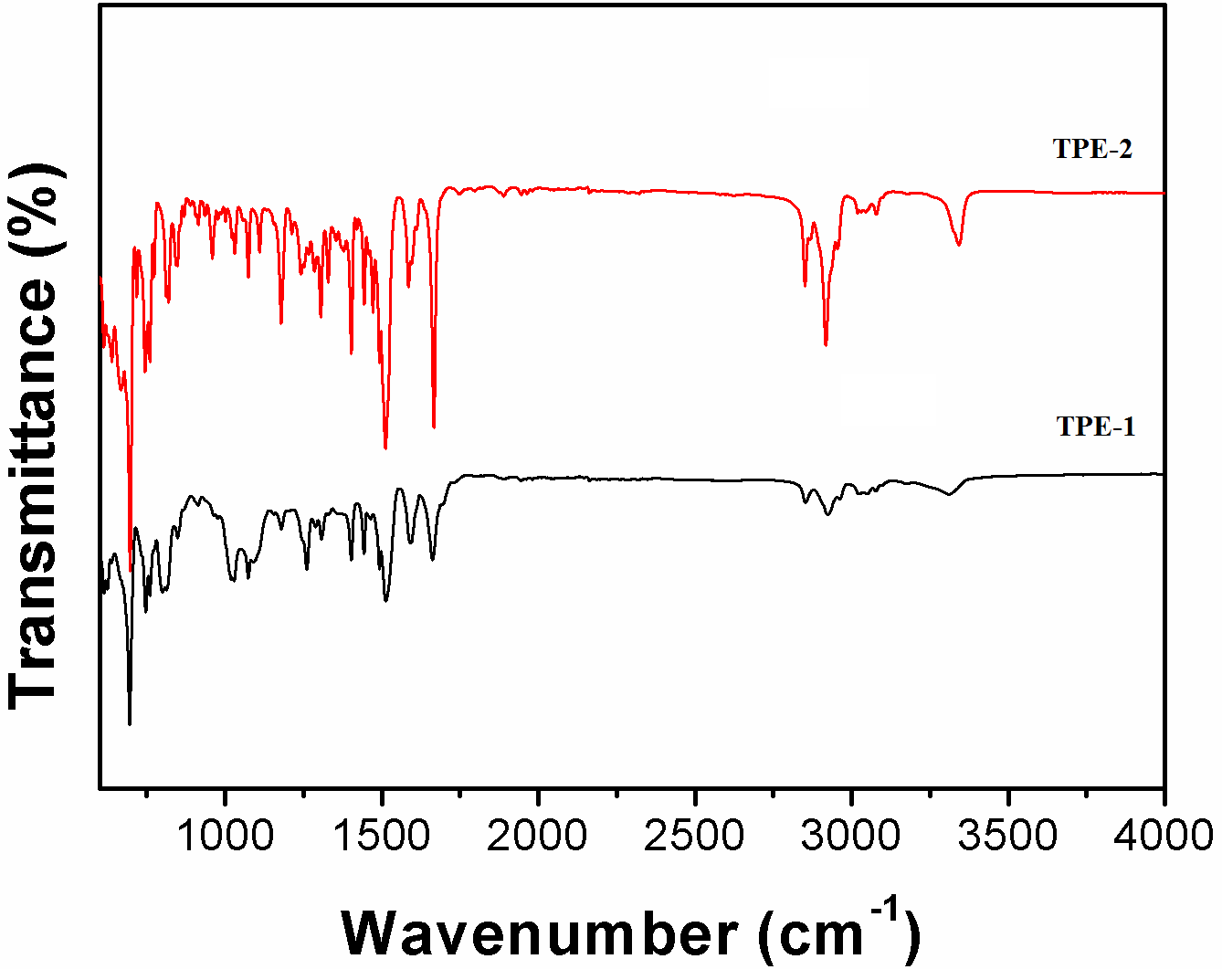

Supplement: Supplementary Information [file srep42898-s1.doc]
